# Microbiota from Exercise Mice Counteracts High-Fat High-Cholesterol Diet-Induced Cognitive Impairment in C57BL/6 Mice

**DOI:** 10.1155/2023/2766250

**Published:** 2023-01-20

**Authors:** Rui Li, Ruitong Liu, Lei Chen, Guiping Wang, Liqiang Qin, Zengli Yu, Zhongxiao Wan

**Affiliations:** ^1^Department of Nutrition and Food Hygiene, School of Public Health, Soochow University, 199 Ren'ai Road, Suzhou 215123, China; ^2^Institute of Nutrition & Health, School of Public Health, Qingdao University, 38 Dengzhou Road, 266021 Qingdao, China; ^3^Laboratory Animal Center, Medical College of Soochow University, 199 Ren'ai Road, Suzhou, China; ^4^College of Public Health, Zhengzhou University, Zhengzhou, 450001 Henan, China

## Abstract

Gut microbes may be the critical mediators for the cognitive enhancing effects of exercise. Via fecal microbiota transplantation (FMT), this study is aimed at determining the mechanism of how voluntary exercise improved learning and memory ability impairment post a high-fat, high-cholesterol (HFHC) diet. The learning and memory abilities assessed via the Morris water maze in the FMT recipient group of voluntary exercising mice were improved compared to sedentary group. 16S rRNA gene sequencing results indicated that exercise-induced changes in gut microbiota distribution were transmissible, mainly in terms of elevated *Lactobacillus*, *Lactobacillus*, and *Eubacterium nodatum*, as well as decreased *Clostrida_UCG-014* and *Akkermansia* after FMT. The neuroprotective effects of FMT were mainly related to the improved insulin signaling pathway (IRS2/PI3K/AKT) and mitochondrial function; inhibition of AQP4; decreased p-Tau at serine 396 and 404; increased BDNF, PSD95, and synaptophysin in the hippocampus; and also decreased HDAC2 and HDAC3 protein expressions in the nuclear and cytoplasmic fractions of the hippocampus. The findings of qRT-PCR suggested that exercise-induced gut microbes, on the one hand, elevated GPR109A and decreased GPR43 and TNF-*α* in the hippocampus. On the other hand, it increased GPR109A and GPR41 expressions in the proximal colon tissue. In addition, total short-chain fatty acid (SCFA), acetic acid, propionic acid, isobutyric acid, valeric acid, and isovaleric acid contents were also elevated in the cecum. In conclusion, exercise-induced alterations in gut microbiota play a decisive role in ameliorating HFHC diet-induced cognitive deficits. FMT treatment may be a new considerable direction in ameliorating cognitive impairment induced by exposure to HFHC diet.

## 1. Introduction

Exercise is gaining great attention as a nonpharmacological approach to maintain brain health and treat neurodegenerative disorders, with numerous mechanisms involved in such as enhancement of brain volume, functional connectivity, and mitochondrial function and reductions in neuroinflammation and oxidative stress [1]. Accumulating evidence began to suggest that gut microbiome might be the key connection between exercise and cognitive function [2–4]. In 2014, Kang et al. [3] demonstrated that forced wheel running enhanced contextual memory, increased the abundance of *Firmicutes*, and decreased abundance of *Bacteroidetes* and *Tenericutes* in C57BL/6 mice; they further reported an association between *Ruminococcaceae* and *Lachnospiraceae* with some fear-conditioning relevant markers [3]. Similarly, 20 weeks of treadmill running improved cognitive functions and histological hallmarks of Alzheimer's disease (AD) in APP/PS1 mice; this was associated with an increased abundance of beneficial short-chain fatty acid- (SCFA-) producing bacteria and decreased microorganism levels involved in disease exacerbation [4]. However, most of the existing findings are correlational; it is still unclear whether the gut microbiota plays a direct role in such exercise-induced cognitive benefits and what are the underlying mechanisms connecting exercise, gut microbiome, and cognitive function.

Fecal microbiota transplantation (FMT), which includes the administration of fecal materials from a healthy donor into another patient's intestinal tract to directly alter the recipient's gut microbiota, might represent a potentially promising approach against cognitive decline and memory dysfunction including AD [5]. For instance, Sun et al. [6] reported that FMT of donor WT mice to APP/PS1 mice prevented AD-like pathology, and this might be associated with improved gut microbiota and SCFAs. In contrast, Kim et al. [7] demonstrated that transplantation of gut microbiota from 5x FAD into normal C57BL/6 mice resulted in colonic inflammation and impaired memory abilities. Likewise, recently, Parker et al. [8] reported that the transfer of aged donor microbiota into young mice accelerated age-associated central nervous system inflammation, and in converse, these detrimental effects were reversed by the transfer of young donor microbiota. Therefore, FMT might represent an effective strategy for clarifying the direct role of gut microbiome in exercise-induced cognitive improvement.

SCFAs, the major end products of microbial fermentation in the gut, have been gaining growing attention as a potential mediator in the pathogenesis of AD [9]. SCFAs might affect the development of AD via multiple mechanisms including maintenance of blood-brain barrier (BBB) integrity, inhibition of neuroinflammation, and regulation of brain metabolism [9]. The effects of SCFAs on brain function are mainly mediated via interactions with G-protein coupled receptors (most notably GPR109A, GPR41, and GPR43) and inhibition of histone deacetylase (HDAC) activity [10]. Additionally, exercise might be a robust mediator for fecal SCFA level. For example, it has been reported that a 6-week moderate to intense aerobic exercise induced a shift in the SCFA-producing bacteria and elevated fecal SCFAs in lean subjects [11]. Also, 6 weeks of voluntary wheel running increased butyrate production and altered fecal bacteriome profiles in C57BL/6 mice [12]. Despite this, whether and how alterations in SCFAs post exercise are functionally involved in the neuroprotective of exercise on host remains obscure.

Here, we aimed to determine whether the cognitive benefit exercise confers on the host mediated through fecal microbiome and its main metabolites (i.e., SCFAs). Specifically, we assessed whether FMT from exercise trained or sedentary donor mice on a normal diet could alter cognitive function, microbiota composition, and fecal SCFAs in sedentary, high-fat, high-cholesterol (HFHC) fed recipients. We further explored the potential mechanisms including insulin signaling, mitochondrial function, neuroinflammation, synaptic function, and HDAC activity in the brain, as well as the gut-brain integrity.

## 2. Materials and Methods

### 2.1. Materials

AIN93-M standard diet was purchased from Trophic Animal Feed High-Tech Co., Ltd. (Nantong, China). A high-fat, high-cholesterol (HFHC, 40% kcal fat and 1.25% kcal cholesterol) was purchased from Dyets Biotechnology Co., Ltd. (Wuxi, China). Reagents for SDS-PAGE were obtained from the Beyotime Institute of Technology (Haimen, China). The molecular weight marker and nitrocellulose membranes for SDS-PAGE were from Bio-Rad (CA, USA). Immobilon Western chemiluminescent HRP substrate (cat#WBKLS0100) was purchased from Millipore (MA, USA). The following antibodies were purchased from Proteintech (MA, USA): insulin receptor substrate 2 (IRS2) (cat#20702-1-AP) and PI3 kinase p110*α* (cat#67071-1-LG). Antiglial fibrillary acidic protein (GFAP) (cat#3670), *β*-actin (cat#66009-1-Ig), histone deacetylase 3 (HDAC3) (cat#3949), phospho-AMPK*α* (Thr172) (cat#2535), and synaptophysin (cat#5461) were from Cell Signaling. Anti-p-Tau ser396 (cat#YP0263), p-Tau ser404 (cat#YP0264), and p-Akt473 (cat#YP006) were from ImmunoWay Biotechnology Company (TX, USA). Brain-derived neurotrophic factor (BDNF) (cat#ab108383) and total OXPHOS (cat#ab110413) were from Abcam (Shanghai, China). Postsynaptic density protein 95 (PSD-95) (cat#AJ1661a) was from abgent. Ib*α*1 (cat#A19776) and HDAC2 (cat#A19626) were from ABclonal Technology Co., Ltd. (Wuhan, China). Horseradish peroxidase-conjugated donkey anti-rabbit secondary antibodies were purchased from Jackson Immuno Research Laboratories (PA, USA). The nylon transfer membrane (cat#RPN303B) was from GE HealthCare (MA, USA). TRNzol Universal Reagent (cat#DP424) was from Tiangen Biotech Co., Ltd. (Beijing, China). PrimeScript™ RT Master Mix (cat#RR036) and SYBR® Premix Ex Taq™ II (cat#RR820) were from Takara (Beijing, China).

### 2.2. Animal Grouping and Interventions

This study involved both donor and recipient groups of male C57BL/6J mice purchased from the GemPharmatech Company (Jiangsu, China) (supplementary Figure 1). All mice were reared in specific pathogen-free conditions with appropriate temperature (20-26°C), humidity (50-60%), and a 12 h light-dark cycle to get adaptability for 1 week before the intervention. Furthermore, water and feed were provided *ad libitum*. The whole procedure abided the Guidelines in the Care and Use of Animals and was approved by the Animal Studies Committee of Soochow University (approval no. SUDA20211206A02).

In stage 1, ten-week-old male C57BL/6J mice (*N* = 16) as donor mice were randomly divided into 2 groups of 8 animals each: the donor sedentary group (DSED) and donor voluntary exercise group (DEX); both were fed with an AIN-93M diet. For the DEX group, mice were housed individually in polypropylene cages with a running wheel (12 cm diameter) and had free and unlimited access to the running wheel for a total of 10 weeks. From weeks 6 to 10, a freshly emptied stool *per* donor mouse was collected every day and pooled with other stools from the same group and stored at -80°C. By performing daily collections of stool samples with the exercise training program progressed, the potential alterations of the microbiota population over the course of the exercise program would be captured, and these will also be transferred to the recipient's mice via gavage to assess potential impacts on brain function readouts.

In stage 2, ten-week-old male C57BL/6J mice (*N* = 24) were randomly divided into 3 groups with 8 animals each: the control group (CON), the recipient-FMT-sedentary-HFHD group (FMTSED), and recipient-FMT-voluntary exercise-HFHC group (FMTEX). The mice were fed with an AIN-93M diet for the CON group and HFHC diet for the FMTSED and FMTEX groups for a total of 12 weeks. In regard to the FMT procedure, firstly, existing microbiota from all 3 groups was depleted by the delivery of 5-day broad-spectrum antibiotic cocktail regime via oral gavage (10 mg/L for ampicillin, metronidazole, and neomycin, 5 mg/L for vancomycin, and 0.1 mg/L for amphotericin B) as described previously by Wang et al. [13] with modifications and used as recipients of FMT. Right before FMT, approximately 1.5 g of donor feces was resuspended in 2 mL sterile PBS; the solution was vigorously mixed for 30 seconds three times before centrifugation at 800 × g for 5 min; then, the crude aqueous fecal extract was collected as described by Zoll et al. [14] with slight modifications. Next, recipient mice from the FMTSED and FMTEX groups were administered with 200 *μ*L of the fresh aqueous fecal extract from the DSED and DEX groups, respectively, two times a week via oral gavage for a total of 12 weeks.

### 2.3. Glucose and Insulin Tolerance Test

After fasting the mice for 12 h by placing them in a new, clean cage with free access to water, glucose tolerance tests (GTT) were performed via intraperitoneal injection (I.P. injection) of glucose (1.25 g/kg body weight). After one-day interval, mice were given an I.P. injection of insulin (1.5 IU/kg body weight) without fasting. Blood glucose levels were measured at the following time intervals: 0, 15, 30, 45, 60, 90, and 120 minutes post injection by tail vein sampling via a glucometer (Roche Diagnostics (Shanghai) Co., Ltd.). The area under the curve (AUC) was calculated by the trapezoidal method to plot the trend in glucose alterations over time.

### 2.4. Behavioral Tests via the Morris Water Maze (MWM)

The experiment included a 5-day navigation trial and a 1-day probe trial to evaluate the learning and memory ability of mice as described previously from our laboratory [15]. In brief, the mice were placed in a circular pool (diameter: 120 cm and height: 40 cm) filled with water (22 ± 1°C). The target event of the navigation trial was that the mice found a hidden platform (1.0 cm below the water surface) within 80 seconds and rested on this platform for 20 seconds. If the platform was not found within 80 seconds, the mice will be gently guided and stayed on the platform for 20 seconds to learn. During the probe trial, the platform was removed and the mice can swim freely in the swimming pool for 80 seconds. To evaluate the spatial learning and memory ability, the time when the mice found the platform (i.e., escape latency) during navigation trial, the number of crossing the platform area, the swimming time and distance, and the proximity in the target quadrant were recorded. Supermaze tracking software (Shanghai XinRuan Information Technology Co., Ltd., Shanghai, China) was used for data collection and analysis.

### 2.5. Tissue Collection and Preservation

The mice were sacrificed with an overnight fast (from 9 : 00 pm to 9 : 00 am) after the behavioral test. The brain was removed, and the parietal-temporal cortex and hippocampus were quickly separated from the whole brain, then frozen in liquid nitrogen, and stored at -80°C. The colon was also obtained and rapidly frozen in liquid nitrogen and then stored at -80°C. Half of all tissues was fixed in 4% paraformaldehyde.

### 2.6. Western Blot Analysis and Nuclear-Cytoplasm Protein Extraction

Total protein was also extracted from the hippocampus of the mice, and proteins were determined by western blotting as described previously by our laboratory [15]. Briefly, protein samples diluted with nonreducing loading buffer were separated alongside prestained molecular size standards in SDS-PAGE gels by electrophoresis and then transferred onto nitrocellulose membranes in cooled transfer buffer. Thereafter, the membrane was covered using nonfat milk for 1 h and incubated at 4°C overnight with respective primary antibodies. The next day, membranes were incubated with secondary antibodies. Proteins were resolved using Immobilon Western Chemiluminescent HRP and exposed to Syngene chemi-imaging system (MD, USA).

Small hippocampal tissues were separated into nuclear and cytoplasmic fractions using NE-PER Nuclear and Cytoplasmic Extraction Reagents as described previously in our laboratory [16]. First, approximately 20 mg of hippocampal tissue was taken into a centrifuge tube; then, precooled CER II solution was added and centrifuged at 16,000 × g for 5 min at 4°C on a bench top high-speed centrifuge. The supernatant (cytoplasmic proteins) was then immediately transferred to a clean precooled tube and stored at -80°C for further western blot analysis. The NER solution was added to the lower precipitated fraction, vortexed vigorously for 15 s, left on ice for 10 min, and repeated 4 times. Then, centrifuge at 4°C, 16,000 × g for 10 min. The supernatant (nuclear proteins) was then transferred to a clean precooled tube and stored in -80°C for further analysis.

### 2.7. Immunofluorescence (IF) Staining for Brain Tissues

Immunofluorescence microscopy was performed on brain sections as described previously from our laboratory [17]. In brief, the slides were blocked with 5% BSA in PBS at room temperature for 30 minutes and then diluted in PBS at 4°C with sheep anti-Ib*α*-1 (1 : 500), rabbit anti-GFAP (1 : 300), and rabbit anti-AQP4 (1 : 500) overnight. After primary antibody culture, the sections were extensively washed with PBS and then cultured with Alexa 488 or Alexa 594 combined with IgG secondary antibody (1 : 200) in the dark at 37°C for 1 hour and then rinsed with PBS three times. DAPI was used for nuclear staining for 10 minutes and then thoroughly washed in distilled water. The sections were then examined using a confocal laser scanning microscope (C2; Nikon, Tokyo, Japan).

### 2.8. Hematoxylin and Eosin (H&E) Staining for Colon Tissues

The colon tissue samples were fixed in 4% paraformaldehyde for 24 hours and then embedded and sliced (5 mm thickness). Paraffin sections were dewaxed with xylene and hydrated with gradient ethanol, and images were obtained using a DM3000 microscope (Phase Contrast, Japan). Villi length was measured by the Image-Pro software (Media Cybernetics, United States).

### 2.9. qRT-PCR Quantification

Total RNA was extracted from mouse hippocampus using TRNzol Universal Reagent according to the manufacturer's protocol. PrimeScript™ RT Master Mix was used for cDNA synthesis from total RNA, and SYBR® Premix Ex Taq™ II was used for real-time quantitative PCR analysis per manufacturer's protocol. Primer sequences used for qRT-PCR were listed in supplementary Table S1.

### 2.10. Quantitative Analysis of Krebs Cycle Intermediate Metabolites by GC-MS

Gas chromatography-mass spectrometry (GC-MS) was used to analyze the Krebs cycle intermediate metabolites as described previously by He et al. [18]. The cerebral cortex was weighed and ground with liquid nitrogen and 10 times the volume of extraction buffer (methanol: chloroform: water = 2 : 5 : 2) was added and vortexed vigorously for 15 seconds. The mixed samples were stored at -80°C overnight. The samples were centrifuged at 4°C for 10 min at 12000 × g in a bench top high-speed centrifuge, and 200 *μ*L of supernatant was freeze-dried under vacuum. Then, 50 *μ*L of methoxylamine (10 mg/mL pyridine solution) was added and incubated at 37°C for 1 h. Finally, 50 *μ*L of BSTFA (1% TMCS, Sigma) was added to the reaction at 60°C for 1 h. The samples were analyzed by Agilent 7890A Weather Chromatography-5975C mass spectrometer. Metabolite content was normalized with an internal standard (myristic acid d27), and a final two-dimensional matrix of metabolite content and sample information was constructed.

### 2.11. Quantification of Short-Chain Fatty Acids in Cecal Content

SCFAs were extracted from feces and analyzed using an Agilent 7890B by GC system (Analytical Technologies, Inc., California, USA), equipped with a flame ionization detector as described previously from our laboratory [15]. Helium gas at 1 ml/min was used as the carrier gas. The column temperature was started at 90°C for 1 min and then increased to 150°C for 1 min, increasing at 15°C per min, then 170°C for 5 minutes, and finally increasing to 240°C for 5 minutes. Samples, all standard compounds, and blanks were injected at an injection volume of 1 *μ*L and an injection temperature of 220°C. OpenLab CDS (Agilent, USA) was used to process the data for peak pickup, standard curve construction, and quantification of SCFAs. Total SCFA is the sum of the concentrations of acetic, propionic, butyric, isobutyric, valeric, and isovaleric acids in the contents of the cecum expressed as *μ*g/g of SCFAs per gram of feces.

### 2.12. Microbiota Analysis

Total bacterial genomic DNA was extracted from all samples using the GHFDE100 DNA isolation kit (GUHE Laboratories, Hangzhou, China), and PCR amplification was performed in the V4 region of the bacterial 16S rRNA gene. Quantitative Insights Into Microbial Ecology 2 (QIIME2) and the R package (v3.2.0) were utilized for the 16S rRNA data analysis. QIIME2 software was used to calculate the alpha diversity index of OTU level, including Chao1, ACE, Shannon, and Simpson indices. The representative sequences of OTU were annotated by QIIME2 based on the SILVA138 database to further generate the OTU list. At each classification level, the community composition of each sample is counted according to the kingdom, phylum, class, order, family, genus, and species. Beta diversity analysis was to calculate the UniFrac distance measure through time software, draw a principal coordinate analysis (PCoA) diagram, and analyze the beta diversity of microbial community structure of different samples. LEfSe (linear discriminant analysis effect size) was performed to detect differentially abundant taxa across groups using the default parameters [19].

### 2.13. Statistical Analyses

Data were expressed as means ± SEM and analyzed with the SPSS version 21 statistical analysis package. Except for data on gut microbiota, the differences among groups were compared by a one-way ANOVA with the Tukey's post hoc test. Statistical significance was set at *p* < 0.05.

## 3. Results

### 3.1. Learning and Memory Ability Test via MWM

**Figure 1:**
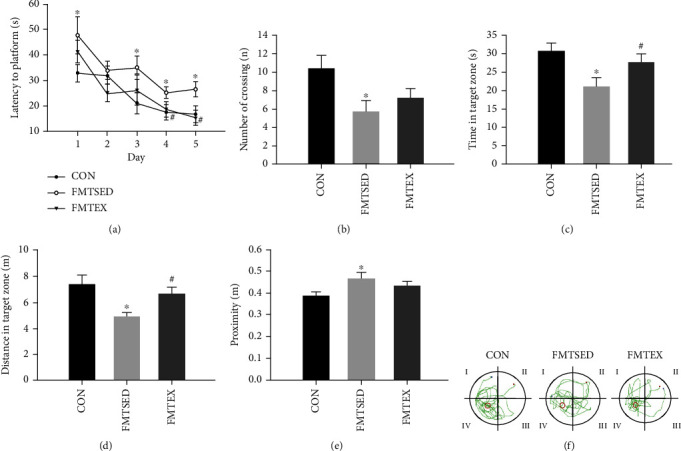
Cognitive function measured via MWM for the C57BL/6J mice. (a) Mean escape latency to hidden platform on the first 5 training days of navigation test. (b) Number of crossing the previous platform location, (c) time spent, (d) swimming distance in the target quadrant, and (e) proximity during probe test. (f) Representative swimming paths of mice from each group. Data were presented as means ± SEM (*N* = 8). ^∗^*p* < 0.05 versus CON and ^#^*p* < 0.05 versus FMTSED.

In the navigation trial, all mice's latency to the platform showed an overall downward trend day by day, indicating that all mice had learned the location of the platform, and the escape latency of mice in the FMTSED group was significantly higher on the first, third, fourth, and fifth days of training when compared to the CON group. Furthermore, the escape latency on days 4 and 5 was substantially lower in the FMTEX group than in the FMTSED group (Figure 1(a)). The number of crossing, time, and distance in the target area was drastically decreased, and the proximity was elevated from the FMTSED group compared to the CON group (Figures 1(b)–1(e)). Compared to the FMTSED group, mice from the FMTEX group spent more time (Figure 1(c)) and traveled farther (Figure 1(d)) in the target area. Moreover, after the platform was removed, the swimming trajectories of mice in the CON and FMTEX groups were primarily concentrated in the target quadrant and had more times crossing the platform, whereas the trajectories of mice in the FMTSED group lacked obvious directivity (Figure 1(f)). It was indicated that mice from fecal transplant of sedentary group had impaired learning and memory ability post-HFHC diet, while fecal transplant from the exercise group was capable of improving HFHC-induced learning and memory dysfunction.

### 3.2. Gut Microbiota Characterization

**Figure 2:**
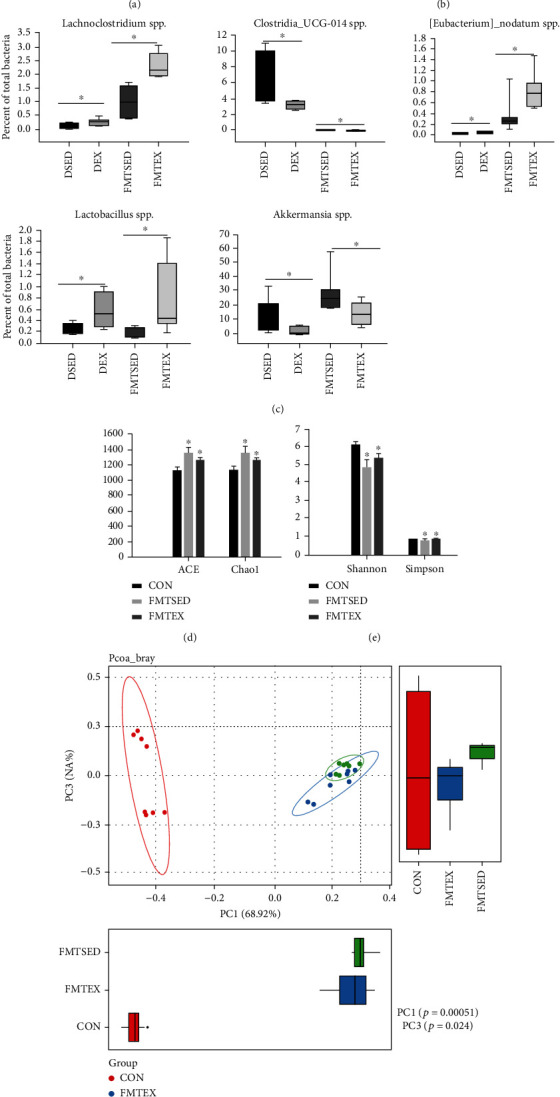
Gut microbial comparisons between donor and recipient mice and microbiota characterization of recipient mice. Relative abundance of the top 10 microbiota determined using 16S rRNA sequences in DSED, DEX, FMTSED, and FMTEX at the phylum (a) and genera (b) levels. (c) Relative abundance (% of total bacteria) of bacterial genera that were differentially represented by the exercise group in donor mice and were remained differentially abundant in recipient mice after transplant. ^∗^*p* < 0.05 versus DSED and FMTSED, respectively. The *α*-diversity indices including ACE, Chao1 (d), Shannon, and Simpson (e) indices, *β*-diversity indices (f), a cladogram of linear discriminant analysis (g) were shown. Data were presented as means ± SEM (*N* = 8). ^∗^*p* < 0.05 versus CON in (d) and (e).

Since all mice used in SED and EX transplant experiments were on the same gut microbiota composition background, thus the cognitive behavior differences between SED and EX recipient mice were mainly due to the transplanted gut microbiota. Therefore, we firstly compared the gut microbiota profiles between the donor and recipient groups to determine whether exercise training-induced changes in the gut microbiota can persist in the recipient mice. The relative abundance of top 10 bacteria at the phylum and genus levels in the DSED and DEX groups of donor mice and the FMTSED and FMTEX groups of recipient mice was similar (Figures 2(a) and 2(b)), suggesting that the donor group intervention induced a differential and transmissible microbial community. Specifically, compared to the recipient control (FMTSED) group, the relative abundance of *Lachnoclostridium*, *Lactobacillus*, and *Eubacterium nodatum* was significantly higher in the recipient exercise (FMTEX) group, *Akkermansia* and *Clostridia_UCG-014* were significantly lower in the FMTEX group, and these trends matched perfectly with the trends between the DSED and DEX groups (Figure 2(c)). The comparison of other bacterial species between the donor and recipient groups is shown in supplementary Table 2. Furthermore, the gut microbial characteristics of donor group mice are shown in supplementary Figure 2.

We then characterized the gut microbiota profiles among CON, FMTSED, and FMTEX groups to determine how the diet and FMT affected the gut microbiota in recipient mice. Compared with the CON group, the ACE and Chao1 indices were significantly higher in the FMTSED and FMTEX groups (Figure 2(d)), while the Shannon and Simpson indices were significantly lower in these two groups (Figure 2(e)). The relative abundance of the top 10 gut-bacterial genera at the phylum, order, phylum, family, and genus levels for the three groups of mice was demonstrated in Table 1. Relative to the CON group, bacterial taxa significantly decreased in the FMTSED group including the phylum *Actinobacteria*, *Firmicutes*, and *Tenericutes*; the class *Erysipelotrichi*, *Coriobacteriia*, *Actinobacteria*, and *Bacilli*; the order *Erysipelotrichales*, *Coriobacteriales*, *Bifidobacteriales*, and *Lactobacillales*; the family *Erysipelotrichaceae*, *Coriobacteriaceae*, and *Clostridiaceae*; and the genus *Lactobacillus* and *Desulfovibrio*. Bacterial taxa including the phylum *Tenericutes*, the class *Coriobacteriia* and *Bacilli*, the order *Coriobacteriales* and *Lactobacillales*, the family *Coriobacteriaceae* and *Clostridiaceae*, and the genus *Lactobacillus* and *Desulfovibrio* were significantly reduced from the FMTEX group compared to the CON group. Bacterial taxa significantly elevated in the FMTSED and FMTEX groups relative to the CON group including the phylum *Cyanobacteria*, *Proteobacteria*, *TM7*, and *Verrucomicrobia*; the class *Verrucomicrobia*, *Deltaproteobacteria*, and *Gammaproteobacteria*; the order *Verrucomicrobiales*, *Desulfovibrionales*, and *Enterobacteriales*; the family *Verrucomicrobiaceae*, *Paraprevotellaceae*, and *Desulfovibrionaceae*; and the genus *Akkermansia*, *Prevotella*, and *Bacteroides*. Relative to the FMTSED group, bacterial taxa significantly elevated in the FMTEX group including the family *Lachnospiraceae* and the genus *Lactobacillus*, while *Verrucomicrobia* at the phylum, class, order, and family levels, *Clostridiaceae* at the family level, and *Akkermansia* at the genus level were significantly reduced. Beta-diversity was further used to detect differences in microbiota composition among groups. As shown in Figure 2(f), PCoA plots showed a clear separation between the FMTSED and FMTEX groups and the CON group, indicating that biodiversity among groups was relatively less affected by FMT and more affected by the diet. The differences in taxonomical terms between the three groups were depicted in Figure 2(g); a total of 80 taxa were significant (LDA > 2.0). Specifically, the bacterial taxa that were increased in the FMTEX group were the phylum *Firmicutes* and *Proteobacteria*, the family *Lachnospiraceae* and *Ruminococcaceae*, and the genus *Prevotella*, *Lactobacillus*, and *Blautia*. These bacteria are all SCFA-producing bacteria.

### 3.3. SCFA Profiles from Cecal Content

As shown in Table 2, total SCFAs and isobutyric acid from the FMTSED group were significantly lower than the CON group, while total SCFAs, acetic acid, propionic acid, isobutyric acid, valeric acid, and isovaleric acid were significantly higher in the FMTEX group than in the CON and FMTSED groups; also, butyric acid from the FMTEX group was high (50.97 ± 22.92 *μ*g/g feces), while those from the CON and FMTSED groups were under detect limitation.

### 3.4. SCFA Receptor mRNA Expression, Colon Histology, and HDAC Protein Expression from Hippocampus

**Figure 3:**
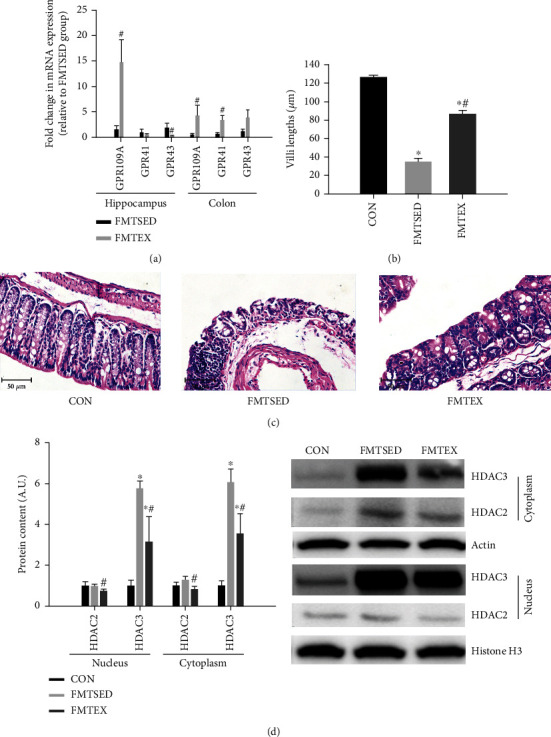
SCFA receptor mRNA expression from both hippocampus and colon, HE staining of colon tissues, and HDAC protein expression from hippocampus. (a) The mRNA expression of GPR41, GPR43, and GPR109A in the hippocampus and proximal colon tissues were measured by qPCR. Colon villi lengths (b) and cross-sections of the mouse colon (c) were shown (H&E stained cross sections; ×20 objective). (d) Protein expression of HDAC2 and HDAC3 in the nuclei and cytosol of hippocampus was measured by western blot. ^∗^*p* < 0.05 versus CON and ^#^*p* < 0.05 versus FMTSED.

To further explore the potential roles of SCFAs in regulating cognitive function, we firstly measured key SCFA receptors mRNA including GPR109A, GPR41, and GPR 43 from both the hippocampus and colon and also determined the colon histology via HE staining. In hippocampal tissues, the mRNA expression of GPR109A and GRP43 in the FMTEX group was dramatically higher and lower than that in the FMTSED group, respectively. In proximal colon tissue, GPR109A and GPR41 mRNA expressions were markedly higher in the FMTEX group than in the FMTSED group (Figure 3(a)). HE staining of colonic tissues showed that the number of inflammatory cells in colonic tissues was significantly lower in the FMTEX group compared with the FMTSED group, and the crypt cells were neatly arranged and not significantly impaired in regular shape. In addition, the length of villi was significantly reduced in the FMTSED and FMTEX groups compared with the control group, while the length of villi was significantly increased in the FMTEX group compared with the FMTSED group (Figures 3(b) and 3(c)). We then measured HDAC2 and HDAC3 protein expressions from both nucleus and cytoplasm because evidence suggests that the effects of SCFA on neurogenesis and neuroprotection may be due to increased neurotrophic factor mRNA levels or promoter activity through inhibition of HDACs [10]. The protein expression of HDAC2 and HDAC3 in the cytoplasm and nucleus fraction of hippocampus from the FMTSED and FMTEX groups was significantly higher than the CON group, while HDAC2 and HDAC3 in both nucleus and cytoplasm from the FMTEX group were significantly decreased compared to the FMTSED group (Figure 3(d)).

### 3.5. Tau Phosphorylation, Synaptic Function, and Neuroinflammation-Related Markers

**Figure 4:**
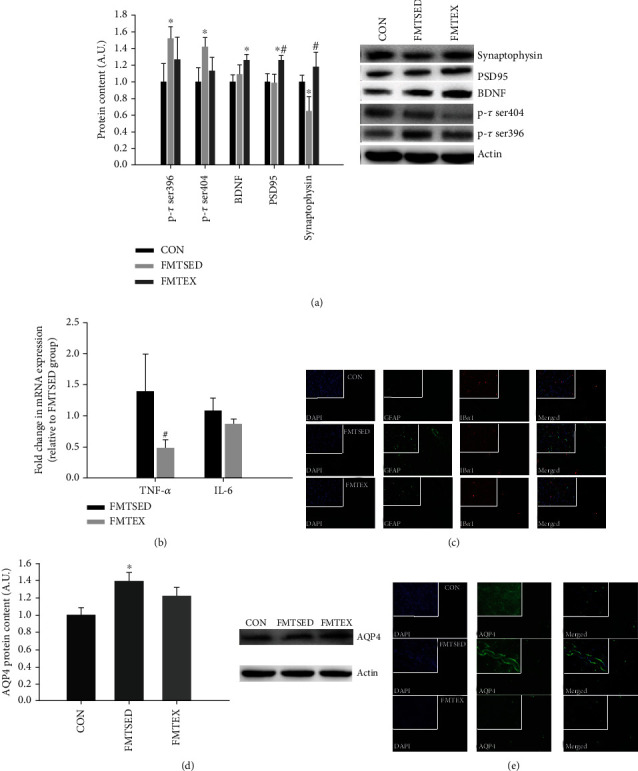
Tau phosphorylation, synaptic function, and neuroinflammation-related markers from hippocampus. (a) Protein expression including p-tau ser396 and 404, BDNF, PSD95, and synaptophysin of hippocampus was measured by western blot. (b) TNF-*α* and IL-6 mRNA expressions from hippocampus were measured by qPCR. (c) Representative double immunofluorescence images of GFAP (in green) and Ib*α*1 (in red) from mouse cerebral cortex (×50 objective). AQP4 protein expression was measured by western blot (d) and immunofluorescence (e) (×50 objective), respectively. Data were presented as means ± SEM (*N* = 8). ^∗^*p* < 0.05 versus CON and ^#^*p* < 0.05 versus FMTSED.

The protein expression of p-tau ser396 and p-tau ser404 was substantially higher, and synaptophysin protein expression was significantly lower in the FMTSED group than the CON group, while BDNF in the FMTEX group was significantly higher compared to the CON group; also, PSD95 and synaptophysin were significantly higher in the FMTEX group than in the FMTSED group (Figure 4(a)). The mRNA expression of TNF-*α* from the FMTEX group was significantly lower compared to the FMTSED group, and there was a downward trend of IL-6 expression in the FMTEX group relative to the FMTSED group (Figure 4(b)). To further evaluate the activation state of immune cells within the brain, we performed double labeling to detect Ib*α*1 and GFAP via immunofluorescence. The numbers of cortical Ib*α*1- and GFAP-positive cells in the FMTSED group were considerably higher than in the CON group (Figure 4(c)). Additionally, we characterized the protein expression of AQP4, which is abundantly expressed on the terminal foot of astrocytes in the periarterial space, and its expression will increase when the body is in an inflammatory state [20, 21]. As shown in Figures 4(d) and 4(e), the protein expression of AQP4 in the FMTSED group was significantly higher than that in the CON group.

### 3.6. Glucose and Insulin Tolerance Test, Insulin Signaling, Krebs Cycle Metabolites, and Mitochondrial Function-Related Markers

**Figure 5:**
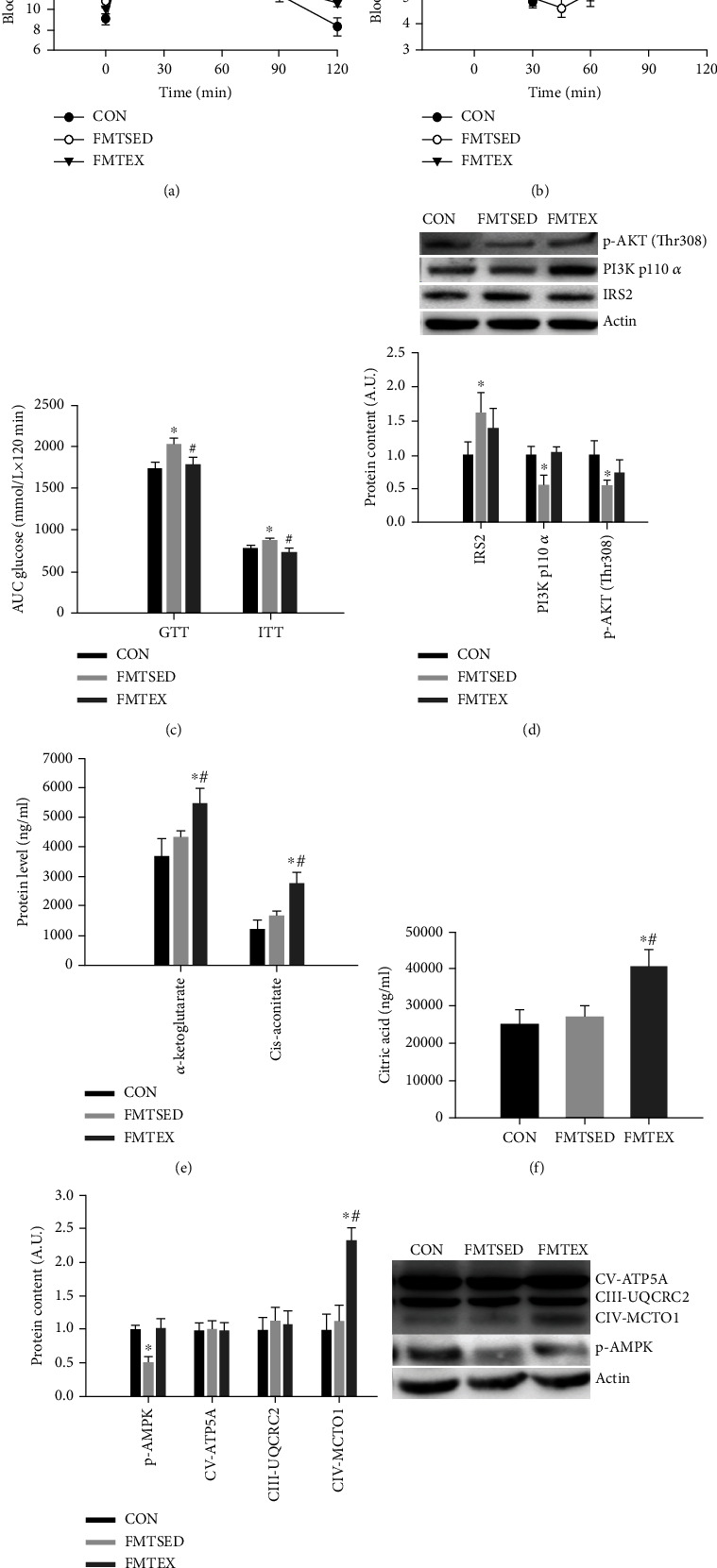
Glucose and insulin tolerance test, hippocampal insulin signaling, and mitochondrial function-related markers, and Krebs cycle metabolites from cerebral cortex. The blood glucose levels at indicated timepoints for GTT (a) and ITT (b), respectively, and (c) AUC of GTT and ITT. (d) IRS2, PI3K p110*α*, and p-AKT (Thr308) protein expressions from hippocampus were measured by western blot. The Krebs cycle intermediate metabolites were measured by GC-MS with *α*-ketoglutarate, cis-aconitate (e), and citric acid (f) as significantly altered metabolites. (g) Protein expression including CV-ATP5A, CIII-UQCRC2, CIV-MTCO1, and p-AMPK from hippocampus was measured by western blot. Data were presented as means ± SEM (*N* = 8). ^∗^*p* < 0.05 versus CON and ^#^*p* < 0.05 versus FMTSED.

During the GTT, the blood glucose level of the FMTSED group at 15, 30, 60, 90, and 120 minutes and the blood glucose level of the FMTEX group at 120 minutes were significantly higher compared to the CON group. Furthermore, the blood glucose level of the FMTEX group was significantly lower at 30, 45, and 120 minutes than that of the FMTSED group (Figure 5(a)). The blood glucose level of the FMTEX group at 0 and 120 minutes was significantly lower than that of the CON and FMTSED groups, respectively, for ITT (Figure 5(b)). The glucose AUC of GTT and ITT was significantly higher in the FMTSED group compared to the CON group, while the glucose AUC of GTT and ITT was significantly lower in the FMTEX group compared to the FMTSED group (Figure 5(c)). In regard to insulin signaling in the hippocampus, the content of IRS-2 from the FMTSED group was notably higher, while the phosphorylation levels of PI3K at p110*α* subunit and Akt at threonine 308 were significantly reduced compared with the CON group (Figure 5(d)), indicating aberrant hippocampal insulin signaling in the FMTSED group. The Krebs cycle is the most important glucose metabolic pathway, supplying neurons with energy closely related to cognitive function [22, 23]. We examined the Krebs cycle intermediate metabolites in mouse prefrontal cortex owing to the limitation of hippocampal tissues. The contents of citric acid, cis-aconitate, and *α*-ketoglutarate were remarkably higher in the FMTEX group compared with the CON group and the FMTSED group (Figures 5(e) and 5(f)). We further measured p-AMPK and OXPHOS complex proteins including CV-ATPA, CIII-UQCRC2, CIV-MTCO1, CII-SDHB, and CI-NDUFB8. Compared with the CON group, p-AMPK expression was dramatically lower in the FMTSED group; CIV-MTCO1 expression level was considerably higher in the FMTEX group compared to the CON and FMTSED groups.

## 4. Discussion

Our study is the very first to provide direct evidence that exercise-induced alterations in intestinal microbiota profiles play a decisive role in ameliorating HFHC diet-induced cognitive impairment at least in C57BL/6 mice. Mechanistically, voluntary exercise increased the relative abundance of beneficial bacteria mainly including *Lactobacillus*, *Lachnoclostridium*, and *Eubacterium nodatum* and decreased the relative abundance of bacteria *Clostridia_UCG-014* and *Akkermansia*, and those alterations were maintained in the recipient group. This was associated with elevated fecal SCFAs, improved intestinal barrier integrity, inhibition of HDAC2 and HDAC3 protein expression and neuroinflammation, improved hippocampal insulin signaling and mitochondrial function, and also restoration of tau phosphorylation in recipient mice.

It has been well established that HFHC diet could lead to cognitive impairment [24, 25]. Existing studies have provided correlational evidence that gut microbiota might play a key role for exercise-induced cognitive improvement [3, 4]. This study found remarkable amelioration of impaired learning and memory function in HFHC diet-fed mice after FMT from the DEX group, which is the very first to confirm that exercise-induced changes in gut microbiota play a decisive role in improving cognitive dysfunction induced by HFHC diet. This study also suggested that FMT treatment may be a new considerable direction in ameliorating cognitive impairment induced by exposure to HFHC diet.

Previous study has reported that the gut microbiota composition of recipient mice is characterized in part by the physical activity status of their respective donor sources, and this difference may be shaped by a group of bacteria that are physiologically relevant and sensitive to exercise training [26]. Our study also confirmed that exercise-induced alterations in gut microbiota are transmissible, and we found that the following exercise-sensitive strains were the main altered and maintained in the recipient mice, i.e., *Lachnoclostridium*, *Lactobacillus*, *Eubacterium nodatum*, *Clostridia_UCG-014*, and *Akkermansia*. Although the function of *Lachnoclostridium* remains controversial, it has been reported to be associated with improved intestinal barrier function and insulin sensitivity post resveratrol intervention [27]. *Lactobacillus* [28] and *Eubacterium nodatum* [29] both are SCFA-producing bacteria. *Clostridia_UCG-014* is a proinflammatory bacterium, mainly accompanied by the accumulation of lipid proinflammatory metabolites [30]. *Akkermansia*, an intestinal symbiont colonizing in the mucosal layer, is a mucin-degrading bacterium of the phylum Verrucomicrobia [31], whose main function is to protect the intestinal barrier [32]. It was indicated that the transmissible bacteria from the EX group mainly included SCFA-producing bacteria (i.e., *Lactobacillus* and *Eubacterium nodatum*) and inflammation-associated bacteria (i.e., *Clostridia_UCG-014* and *Akkermansia*). Furthermore, it should be realized that HFHC diet also greatly affected the gut microbiota profiles of the recipient mice, as shown by the differences in *α*-diversity and *β*-diversity among the CON, FMTSED, and FMTEX groups. However, those did not affect the decisive role of exercise-induced gut microbiota in the maintenance of organismal brain health after FMT.

Existing evidence suggests that SCFAs might be the key connections between gut microbiota and cognitive function [9]. Our study also provided direct evidence that SCFAs might be critical for improving HFHC-induced cognitive impairment post FMT from the DEX group. We firstly observed that total SCFAs and all specific SCFAs (i.e., acetic acid, propanoic acid, butyric acid isobutyric acid, valeric acid, and isovaleric acid) were all significantly increased from the FMTEX group compared to the FMTSED group. We then observed that the FMTEX group had elevated GPR109A mRNA from hippocampus and elevated GPR109A, GPR41, and GPR43 mRNA expressions from the colon. Additionally, HDAC2 and HDAC3 protein expressions were inhibited from the FMTEX group compared to the CON group. Previously, it has been reported that exercise inhibited HDAC2 and HDAC3 mRNA expressions, and the binding of both HDAC2 and HDAC3 to the *Bdnf* promoter was decreased post exercise [33]. Also, BDNF can upregulate the expression of PSD95 and synaptophysin [34, 35]. It is likely that elevated BDNF, PSD95, and synaptophysin protein expression post FMT from the DEX group might be owing to the elevated SCFAs and its subsequent inhibition on HDAC2 and HDAC3. Additionally, evidence also suggests that SCFAs can interact with a variety of immune cells, influencing peripheral inflammation [36] and also directly affecting microglia structural and functional integrity [37], thereby potentially affecting neuroinflammation [10]. Consistently, we found that FMT from the DEX group improved the impaired gut barrier integrity and overactivated neuroinflammation state post HFHC. Collectively, it was indicated that FMT from the DEX group may improve HFHC diet-induced cognitive dysfunction by elevating SCFA-producing bacteria and other transmissible bacteria and increasing fecal SCFA level, consequently improving intestinal stability, synaptic plasticity-related proteins, and neuroinflammation.

Disordered brain insulin signaling also plays a role in learning and memory impairment [38]. Mitochondrial dysfunction plays a significant early impact on AD pathology [39]. Interestingly, evidence also reported that the mitochondria-microbiota cross-talk existed in neurodegenerative diseases. For example, gut microbiota generate SCFAs that can not only be used as fuels sources but also serve to regulate mitochondrial function; conversely, mitochondria can impact the number and types of bacteria colonizing the gut [40]. In the present study, FMT from the DEX group was capable of improving peripheral glucose and insulin tolerance and hippocampal abnormal insulin signaling post HFHC. We further found that important intermediate metabolites of the Krebs cycle including *α*-ketoglutarate, cis-aconitate, and citric acid and one of OXPHOS complex proteins, i.e., cytochrome c oxidase subunit 1 of complex IV (CIV-MCTO1), were elevated, and p-AMPK was restored to normal levels after FMT from the DEX group. Additionally, we demonstrated that FMT from the DEX group was capable of restoring HFHC-induced abnormal tau protein phosphorylation. Overall, improved insulin signaling, mitochondrial function, and restored tau protein phosphorylation post FMT from the DEX group might also be involved in ameliorating HFHC diet-induced cognitive dysfunction, whereas further studies are required to clarify the connections between gut microbiota, SCFA level, and brain insulin signaling and mitochondrial function post FMT from the DEX group.

## 5. Conclusion

Our study demonstrated that exercise-induced alterations in the gut microbiota were transmissible and could improve HFHC diet-induced cognitive impairment and that the transmissible bacteria post exercise was mainly characterized by SCFA-producing bacteria and inflammation-associated bacteria. We also demonstrated that FMT from the DEX group had elevated fecal SCFA levels, inhibited HDAC2 and HDAC3 protein expression and neuroinflammation, and improved synaptic plasticity, insulin signaling, mitochondrial function, and tau phosphorylation from hippocampus. Our results also provide a rational for FMT from the exercise group to restore brain plasticity and eventually to counteract the devastating side effects of prolonged HFHC diet exposure.

## Figures and Tables

**Table 1:** The relative abundance of the top 10 gut bacterial genera at the phylum, class, order, family, and genus levels (%) (mean ± SEM).

Level	CON	FMTSED	FMTEX
*Phylum*			
Actinobacteria	0.0678 ± 0.0200	0.0111 ± 0.0033^∗^	0.0225 ± 0.0091
Cyanobacteria	0.0000 ± 0.0000	0.0003 ± 0.0001^∗^	0.0001 ± 0.0000^∗^
Firmicutes	0.4230 ± 0.0410	0.2351 ± 0.0394^∗^	0.3255 ± 0.0449
Proteobacteria	0.0178 ± 0.0098	0.0790 ± 0.0051^∗^	0.0916 ± 0.0094^∗^
TM7	0.0000 ± 0.0000	0.0007 ± 0.0005	0.0001 ± 0.0000^∗^
Tenericutes	0.0015 ± 0.0004	0.0002 ± 0.0001^∗^	0.0003 ± 0.0002^∗^
Verrucomicrobia	0.0456 ± 0.0107	0.2872 ± 0.0522^∗^	0.1446 ± 0.0271^∗^^#^
*Class*			
Verrucomicrobia	0.0456 ± 0.0107	0.2872 ± 0.0522^∗^	0.1446 ± 0.0271^∗^^#^
Erysipelotrichi	0.1010 ± 0.0189	0.0366 ± 0.0100^∗^	0.0836 ± 0.0241
Deltaproteobacteria	0.0173 ± 0.0100	0.0619 ± 0.0056^∗^	0.0620 ± 0.0068^∗^
Coriobacteriia	0.0506 ± 0.0185	0.0031 ± 0.0006^∗^	0.0036 ± 0.0009^∗^
Gammaproteobacteria	0.0004 ± 0.0001	0.0170 ± 0.0065^∗^	0.0294 ± 0.0037^∗^
Actinobacteria	0.0172 ± 0.0024	0.0080 ± 0.0032^∗^	0.0200 ± 0.0090
Bacilli	0.0233 ± 0.0015	0.0048 ± 0.0008^∗^	0.0104 ± 0.0003^∗^
*Order*			
Verrucomicrobiales	0.0456 ± 0.0107	0.2872 ± 0.0522^∗^	0.1446 ± 0.0271^∗^^#^
Erysipelotrichales	0.1010 ± 0.0189	0.0366 ± 0.0100^∗^	0.0836 ± 0.0241
Desulfovibrionales	0.0173 ± 0.0100	0.0619 ± 0.0056^∗^	0.0620 ± 0.0068^∗^
Coriobacteriales	0.0506 ± 0.0185	0.0031 ± 0.0006^∗^	0.0036 ± 0.0009^∗^
Enterobacteriales	0.0004 ± 0.0001	0.0170 ± 0.0065^∗^	0.0294 ± 0.0037^∗^
Bifidobacteriales	0.0172 ± 0.0024	0.0080 ± 0.0032^∗^	0.0200 ± 0.0090
Lactobacillales	0.0233 ± 0.0015	0.0048 ± 0.0008^∗^	0.0104 ± 0.0003^∗^
*Family*			
Verrucomicrobiaceae	0.0456 ± 0.0107	0.2872 ± 0.0522^∗^	0.1446 ± 0.0271^∗^^#^
Erysipelotrichaceae	0.1010 ± 0.0189	0.0366 ± 0.0100^∗^	0.0836 ± 0.0241
Paraprevotellaceae	0.0000 ± 0.0000	0.0735 ± 0.0146^∗^	0.0965 ± 0.0301^∗^
Lachnospiraceae	0.0417 ± 0.0060	0.0395 ± 0.0100	0.0590 ± 0.0051^∗^^#^
Desulfovibrionaceae	0.0173 ± 0.0100	0.0619 ± 0.0056^∗^	0.0620 ± 0.0068^∗^
Coriobacteriaceae	0.0506 ± 0.0185	0.0031 ± 0.0006^∗^	0.0036 ± 0.0009^∗^
Clostridiaceae	0.0154 ± 0.0034	0.0055 ± 0.0015^∗^	0.0028 ± 0.0015^∗^^#^
*Genus*			
Akkermansia	0.0456 ± 0.0107	0.2872 ± 0.0522^∗^	0.1446 ± 0.0271^∗^^#^
Prevotella	0.0000 ± 0.0000	0.0735 ± 0.0146^∗^	0.0965 ± 0.0301^∗^
Bacteroides	0.0001 ± 0.0000	0.0205 ± 0.0068^∗^	0.0166 ± 0.0060^∗^
Lactobacillus	0.0218 ± 0.0013	0.0021 ± 0.0003^∗^	0.0071 ± 0.0023^∗^^#^
Desulfovibrio	0.0173 ± 0.0098	0.0000 ± 0.0000^∗^	0.0000 ± 0.0000^∗^

^∗^ *p* <0.05 versus CON group by Mann–Whitney *U* test. ^#^*p* <0.05 versus FMTSED group by Mann–Whitney *U* test.

**Table 2:** The content of short-chain fatty acids in recipient mice feces (*μ*g/g) (mean ± SEM).

	CON	FMTSED	FMTEX
Total SCFA	842.65 ± 104.08	525.35 ± 79.65^∗^	1574.09 ± 287.63^∗^^#^
Acetic acid	504.21 ± 59.60	370.12 ± 56.09	654.06 ± 35.65^∗^^#^
Propanoic acid	133.24 ± 20.48	70.63 ± 23.29	269.53 ± 73.05^∗^^#^
Butyric acid	UD	UD	50.97 ± 22.92
Isobutyric acid	205.73 ± 28.90	68.22 ± 9.16^∗^	256.89 ± 67.94^∗^^#^
Valeric acid	0.39 ± 0.34	3.21 ± 2.29	221.83 ± 92.52^∗^^#^
Isovaleric acid	UD	13.17 ± 9.50	120.81 ± 44.91^∗^^#^

^∗^ *p* <0.05 versus CON group by one-way ANOVA. ^#^*p* <0.05 versus FMTSED group by one-way ANOVA. UD, under detect limitation.

## Data Availability

All data generated or analyzed during this study are included in this published article and its supplementary information files.

## Supplementary Materials

Supplementary Materials Figure S1: experimental overview. Figure S2: gut microbiota characterization of donor mice. Table S1: the primer sequences for real-time qPCR. Table S2: the relative abundance of gut bacterial genera at the phylum, class, order, family, and genus levels (%) (means ± SEM).
