# Supplementary material for: Microbiota from Exercise Mice Counteracts High-Fat High-Cholesterol Diet-Induced Cognitive Impairment in C57BL/6 Mice
Source: Oxid Med Cell Longev. 2023 Jan 20;2023:2766250. doi: 10.1155/2023/2766250 (PMC9883105; doi:10.1155/2023/2766250)
Supplement: Supplementary Materials — Figure S1: experimental overview. Figure S2: gut microbiota characterization of donor mice. Table S1: the primer sequences for real-time qPCR. Table S2: the relative abundance of gut bacterial genera at the phylum, class, order, family, and genus levels (%) (means ± SEM). [file 2766250.f1.zip › FMT supplementary figure legends.docx]

**Figure S1 Experimental overview.**

In stage1, donor mice were fed with normal diets and divided into DSED and DEX group, with a freshly emptied stool *per* donor mouse was collected every day from week 6 to 10 and stored at - 80°C. In stage 2, existing microbiota from all 3 groups were depleted by the delivery of 5-day-broad-spectrum antibiotic cocktail regime. Recipient mice from FMTSED and FMTEX groups were administered with 200 μL of the fresh aqueous fecal extract from DSED and DEX groups, respectively two times a week via oral gavage for a total of 12 weeks.

**Figure S2 Gut microbiota characterization of donor mice.**

The α diversity indices including ACE, Chao1 (A), Shannon, and Simpson (B) index, β diversity indices (C) and Linear discriminant analysis, LDA score (D) of DSED and DEX. Data were presented as means ± SEM (N=8). * p < 0.05 versus DSED.
