# Supplementary material for: Microbiota from Exercise Mice Counteracts High-Fat High-Cholesterol Diet-Induced Cognitive Impairment in C57BL/6 Mice
Source: Oxid Med Cell Longev. 2023 Jan 20;2023:2766250. doi: 10.1155/2023/2766250 (PMC9883105; doi:10.1155/2023/2766250)
Supplement: Supplementary Materials — Figure S1: experimental overview. Figure S2: gut microbiota characterization of donor mice. Table S1: the primer sequences for real-time qPCR. Table S2: the relative abundance of gut bacterial genera at the phylum, class, order, family, and genus levels (%) (means ± SEM). [file 2766250.f1.zip › FMT supplementary tables.docx]

**Table S1 The primer sequences for real-time qPCR.**

| **Genes** | **sequences** |
| --- | --- |
| 18s | F:5’- GCAAGACGGACCAGAGCG-3’ |
|  | R:5’- GTGGTGCCCTTCCGTCAA-3’ |
| GPR109A | F:5’- CTGGAGGTTCGGAGGCATC -3’ |
|  | R:5’- TCGCCATTTTTGGTCATCATGT -3’ |
| GPR42 | F:5’- CTTCTTTCTTGGCAATTACTGGC -3’ |
|  | R:5’- CCGAAATGGTCAGGTTTAGCAA -3’ |
| GPR43 | F:5’- CTTGATCCTCACGGCCTACAT-3’ |
|  | R:5’- CCAGGGTCAGATTAAGCAGGAG-3’ |
| ICAM1 | F:5’- GTGATGCTCAGGTATCCATCCA -3’ |
|  | R:5’- CACAGTTCTCAAAGCACAGCG-3’ |
| TNF-α | F:5’- CCCTCACACTCAGATCATCTTCT -3’ |
|  | R:5’- GCTACGACGTGGGCTACAG -3’ |
| IL-6 | F:5’- TAGTCCTTCCTACCCCAATTTCC -3’ |
|  | R:5’- TTGGTCCTTAGCCACTCCTTC -3’ |

**Table S2 The relative abundance of gut bacterial genera at the phylum, class, order, family and genus level (%) (Mean ± SEM)**

| **level** | **D-CON** | **D-EX** | **FMTSED** | **FMTEX** |
| --- | --- | --- | --- | --- |
| **phylum** |  |  |  |  |
| Verrucomicrobia | 0.1384 ± 0.0476 | 0.0260 ± 0.0103* | 0.2872 ± 0.0522 | 0.1446 ± 0.0271** |
| **class** |  |  |  |  |
| Verrucomicrobiae | 0.1384 ± 0.0478 | 0.0026 ± 0.0111* | 0.2872 ± 0.0522 | 0.1446 ± 0.0271** |
| **order** |  |  |  |  |
| Clostridiales | 0.0004 ± 0.0000 | 0.0001 ± 0.0000* | 0.0017 ± 0.0004 | 0.0197 ± 0.0020** |
| Clostridia_UCG-014 | 0.0730 ± 0.0130 | 0.0327 ± 0.0020* | 0.0005 ± 0.0001 | 0.0001 ± 0.0001** |
| Peptostreptococcales-Tissierellales | 0.0009 ± 0.0001 | 0.0014 ± 0.0002* | 0.0107 ± 0.0022 | 0.0172 ± 0.0018** |
| Verrucomicrobiales | 0.1384 ± 0.0478 | 0.0260 ± 0.0111* | 0.2872 ± 0.0522 | 0.1446 ± 0.0271** |
| **family** |  |  |  |  |
| Eggerthellaceae | 0.0027 ± 0.0008 | 0.0078 ± 0.0013* | 0.0004 ± 0.0001 | 0.0009 ± 0.002** |
| Clostridia_UCG-014 | 0.0730 ± 0.0130 | 0.0328 ± 0.0021* | 0.0005 ± 0.0001 | 0.0001 ± 0.0001** |
| Lachnospiraceae | 0.1273 ± 0.0140 | 0.1543 ± 0.0177* | 0.0395 ± 0.0100 | 0.0590 ± 0.0051** |
| Streptococcaceae | 0.0001 ± 0.0000 | 0.0001 ± 0.0000* | 0.0001 ± 0.0000 | 0.0005 ± 0.0000** |
| Anaerovoracaceae | 0.0009 ± 0.0001 | 0.0014 ± 0.0002* | 0.0066 ± 0.0021 | 0.0121 ± 0.0019** |
| Akkermansiaceae | 0.1384 ± 0.0478 | 0.0260 ± 0.0111** | 0.2872 ± 0.0522 | 0.1446 ± 0.0271** |
| **genus** |  |  |  |  |
| Christensenellaceae_uncultured | 0.0002 ± 0.0059 | 0.0004 ± 0.0000* | 0.0000 ± 0.0000 | 0.0001 ± 0.0000** |
| Oscillospiraceae_NK4A214 | 0.0012 ± 0.0000 | 0.0019 ± 0.0000* | 0.0044 ± 0.0000 | 0.0091 ± 0.0000** |

* *p*<0.05 versus CON group by Mann-Whitney U test

** *p*<0.05 versus FMTSED group by Mann-Whitney U test
