# Supplementary figures and images for: Microbiota from Exercise Mice Counteracts High-Fat High-Cholesterol Diet-Induced Cognitive Impairment in C57BL/6 Mice
Source: Oxid Med Cell Longev. 2023 Jan 20;2023:2766250. doi: 10.1155/2023/2766250 (PMC9883105; doi:10.1155/2023/2766250)

## Stage1

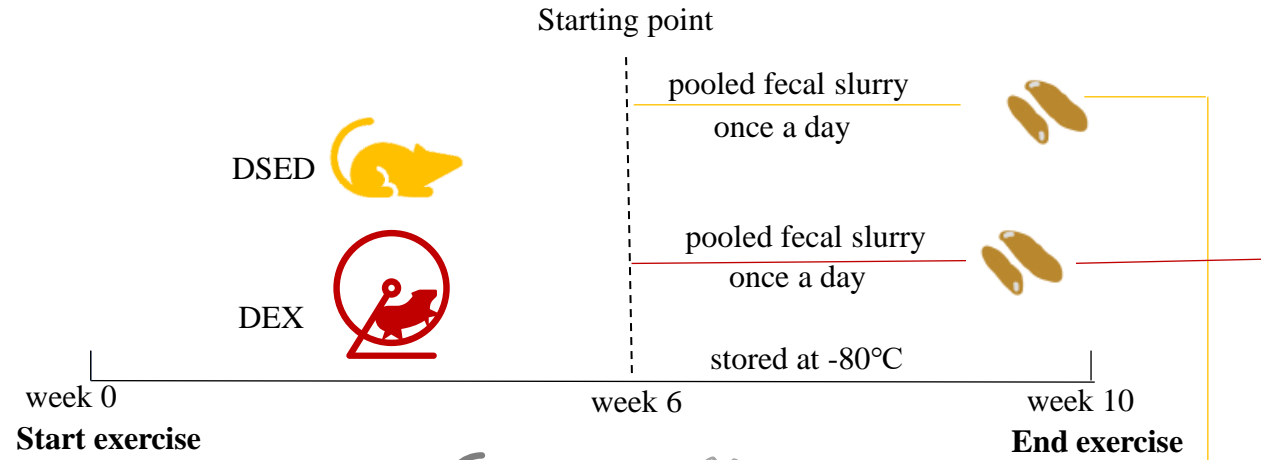

## Stage2

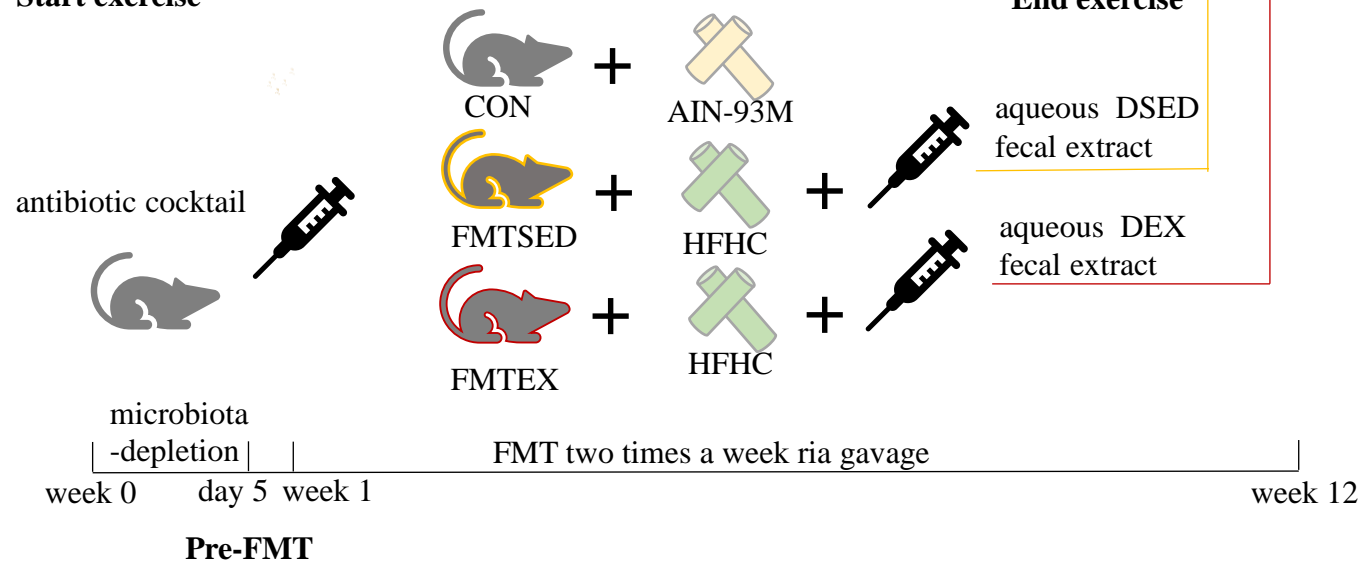

Supplement: Supplementary Materials — Figure S1: experimental overview. Figure S2: gut microbiota characterization of donor mice. Table S1: the primer sequences for real-time qPCR. Table S2: the relative abundance of gut bacterial genera at the phylum, class, order, family, and genus levels (%) (means ± SEM). [file 2766250.f1.zip › supplementary figure1.pdf]

A

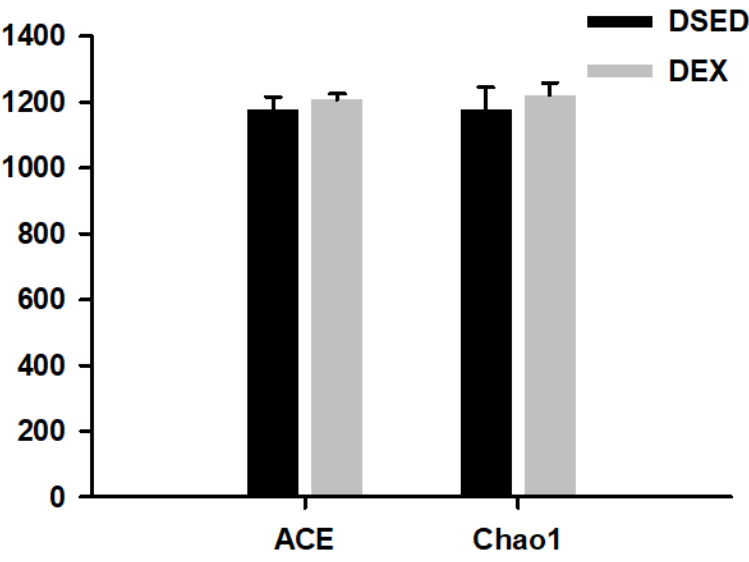

B

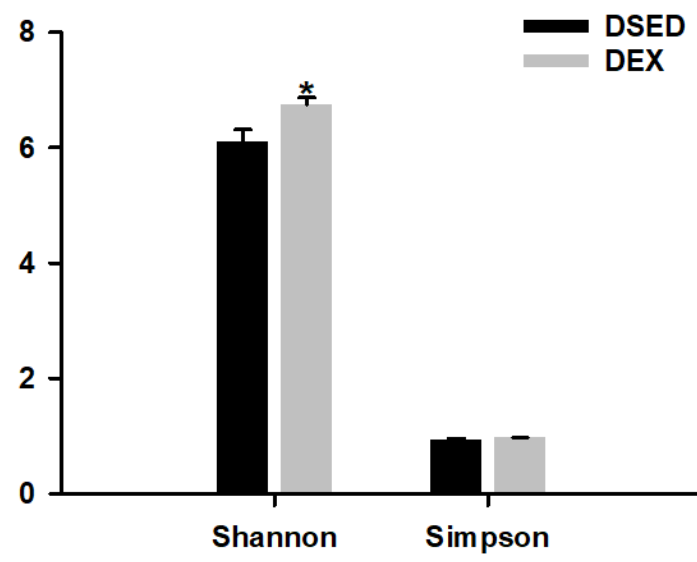

C

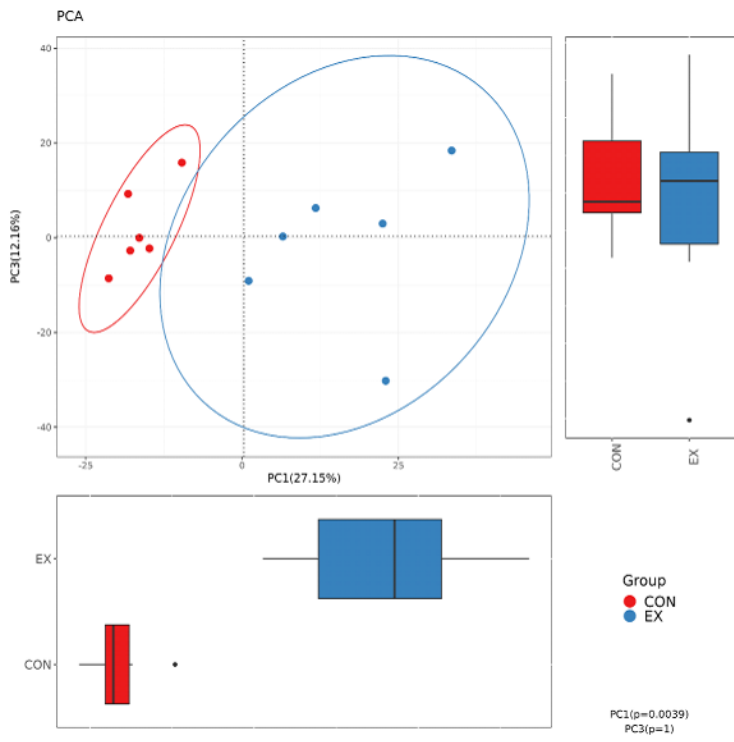

D

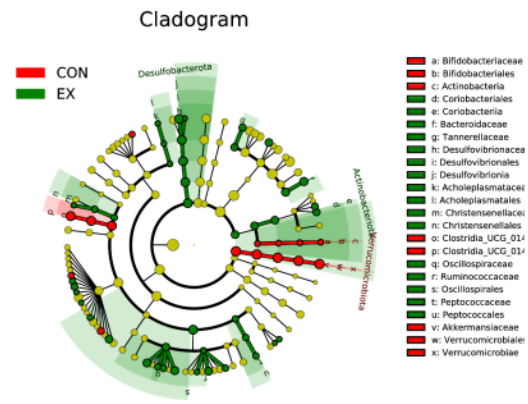

Supplement: Supplementary Materials — Figure S1: experimental overview. Figure S2: gut microbiota characterization of donor mice. Table S1: the primer sequences for real-time qPCR. Table S2: the relative abundance of gut bacterial genera at the phylum, class, order, family, and genus levels (%) (means ± SEM). [file 2766250.f1.zip › supplementary figure2.PDF]
